# Predictive value of the preoperative prognostic nutritional index for postoperative progression in patients with pancreatic neuroendocrine neoplasms

**DOI:** 10.3389/fnut.2022.945833

**Published:** 2022-09-08

**Authors:** Mengfei Fu, Li Yu, Liu Yang, Yang Chen, Xiao Chen, Qinyu Hu, Hui Sun

**Affiliations:** ^1^Department of Endocrinology, Union Hospital, Tongji Medical College, Huazhong University of Science and Technology, Wuhan, China; ^2^Hubei Provincial Clinical Research Center for Diabetes and Metabolic Disorders, Wuhan, China; ^3^Department of Emergency Medicine, Union Hospital, Tongji Medical College, Huazhong University of Science and Technology, Wuhan, China

**Keywords:** prognostic nutritional index, pancreatic neuroendocrine neoplasms, nutritional status, progression-free survival, prognosis

## Abstract

**Objective:**

The preoperative nutritional status of cancer patients is closely related to prognosis. The prognostic nutritional index (PNI) has been shown to predict the prognosis of a variety of tumors, but its study in pancreatic neuroendocrine neoplasms (pNENs) is lacking. The aim of the present study is to investigate the predictive value of the preoperative PNI for postoperative progression in patients with pNENs.

**Methods:**

The medical records of 181 patients with pNENs, who underwent surgery, were retrospectively analyzed. A time-dependent receiver operating characteristic (ROC) curve was plotted to determine the optimal cut-off value of the preoperative PNI. Correlations between the preoperative PNI and clinicopathological parameters were analyzed using multiple linear regression. A Kaplan-Meier curve was applied to assess the progression-free survival (PFS) rate, which was tested using a log rank. Univariate and multivariate Cox proportional risk regression models were used to analyze the predictive value of the preoperative PNI on prognosis.

**Results:**

The optimal cut-off value of the preoperative PNI was 48.275. The patients were divided into a high PNI group (PNI > 48.275, *n* = 92) and a low PNI group (PNI ≤ 48.275, *n* = 89). The proportion of patients with tumor progression after surgery was significantly higher in the low PNI group compared with that in the high PNI group (*P* = 0.004). The Kaplan-Meier curve showed that the PFS rate after surgery was significantly lower in the low PNI group compared with that in the high PNI group (*P* = 0.026). The preoperative PNI was an independent predictor of PFS (HR: 2.727, 95% CI: 1.174∼6.333, *P* = 0.020).

**Conclusion:**

The preoperative PNI has a predictive value for postoperative progression in patients with pNENs.

## Introduction

Pancreatic neuroendocrine neoplasms (pNENs) are a group of rare heterogeneous tumors originating from pancreatic neuroendocrine cells, accounting for approximately 2% of pancreatic tumors (1, 2). The slow growth rate of the tumor and the lack of a specific clinical presentation make the diagnosis of this disease more difficult. With the widespread use of high-quality imaging techniques, the incidence of this disease is increasing every year (3, 4). Currently, surgery occupies an important place in the management of this disease (5–8). However, tumor recurrence and metastasis can still be observed during patients’ follow-up after surgery, which reflects a poor prognosis. Like other tumors, the grading and staging of pNENs are crucial for the prognostic assessment of patients. In addition, clinicopathological characteristics such as age, gender, race, tumor size, and location, have been shown to be closely related to overall survival (OS) (9). The study by Landoni et al. found that the functional status of the tumor, lymph node status, tumor grade, and vascular infiltration are independent predictors of disease progression in pNENs (10). However, due to individual differences and tumor heterogeneity, these prognostic factors have limitations in practical clinical application (11), and therefore, the predictors of postoperative progression of pNENs still need to be investigated in depth.

It has been found that the nutritional status is closely related to disease prognosis and is particularly significant for oncology patients (12, 13). For surgery patients, there is a significant statistical correlation between preoperative malnutrition and poor postoperative wound healing, the increased incidence of complications (14, 15). The prognostic nutritional index (PNI) is an index used to assess the nutritional and immune statuses of surgical patients that can be calculated from serum albumin levels and total peripheral blood lymphocyte count. Compared with other nutritional assessment tools, this index has the advantage of being non-invasive and simple to calculate. Therefore, it has been widely used in the prognostic assessment of various diseases such as cancer, adverse cardiovascular events, cerebrovascular accidents, and in the treatment and management of diseases (16–19). Several existing meta-analyses have shown that cancer patients with low PNI have lower postoperative OS, recurrence-free survival, and PFS compared with those in patients with high PNI (20–22). This suggests that low PNI is a risk factor affecting the postoperative prognosis of cancer patients, and PNI can be used to assess the postoperative prognosis of cancer patients.

However, although the use of PNI has become more popular in assessing the prognosis of tumors in various organs, no study has yet analyzed the association between preoperative PNI and postoperative progression of pNENs. This indicates that there is still a gap in this research field as to whether preoperative PNI can be used as a predictor of postoperative progression of pNENs. Therefore, to clarify this question, we conducted the present study.

## Materials and methods

### Study population and data collection

Patients who underwent surgery and had pathologically confirmed pNENs from April 2009 to September 2021 at Union Hospital, Tongji Medical College, Huazhong University of Science and Technology, Wuhan, China, were retrospectively included. The clinicopathological characteristics of patients were collected from the hospital’s electronic medical record system, including age, gender, residence, height, weight, and personal history (whether smoking or drinking). The date of surgery was recorded, and routine blood test and blood biochemical indices such as total and percentage of peripheral blood lymphocytes, hemoglobin, total serum protein, serum albumin, triglycerides, and cholesterol within a week before surgery, were summarized. Following these procedures, the patients were followed up. Patients with exocrine pancreatic malignancy and hereditary diseases such as multiple endocrine neoplasia type 1 (MEN1), neurofibromatosis type 1 (NF1), and too many missing items in the medical records, and missed visits, were excluded.

The location of the tumor was divided into the head or neck and the body or tail of pancreas. Tumors were classified as functional and non-functional, based on whether they could secrete substances, such as peptide hormones or biogenic amines, and produce the corresponding clinical symptoms. The tumor grading and staging were performed using the grading criteria in the 2019 World Health Organization (WHO) classification of endocrine organ tumors and the 8th American Joint Committee on Cancer (AJCC) TNM staging criteria for pancreatic neuroendocrine tumors, respectively (23, 24).

Places where patients lived continuously for more than 10 years were defined as residence and were divided into urban and rural groups. Patients were classified into a smoking or drinking group if they had smoked (≥10 cigarettes/day) or consumed alcohol (≥100 ml/day) for more than 3 years prior to admission. The body mass index (BMI) was defined as weight (kg)/height squared (m^2^). For the Chinese population, the reference range of normal BMI was 18.5∼23.9 kg/m^2^. Preoperative PNI was calculated from preoperative serological indicators (serum albumin level and total peripheral blood lymphocyte count) as PNI = serum albumin (g/L) + 5 × total peripheral blood lymphocyte count (*10^9^/L).

### Follow-up

The patients who were included in the study were followed up by telephone or through outpatient visits, with an endpoint event defined as any form of tumor progression such as recurrence and metastasis, or patient death from any cause, with a follow-up deadline of March 2022. Progression-free survival (PFS) was defined as the time between the day of the patient’s surgery and the occurrence of the endpoint event.

### Statistical analysis

For continuous variable data, the Kolmogorov-Smirnov test was used to analyze whether the data conformed to a normal distribution, and if so, the mean ± standard deviation (SD) was used, and vice versa, the median M (P_25_, P_75)_ was used. Categorical information was expressed using numbers and percentages (n, %). The time-dependent receiver operating characteristic (ROC) curve was plotted and the Youden index was calculated to find the best cut-off value for preoperative PNI. The independent-samples *t*-test, the non-parametric rank sum test, and the chi-square test were used for comparison between groups, respectively, when applicable. Multiple linear regression was used to analyze the correlation between preoperative PNI and clinicopathological parameters. The Kaplan-Meier curve and the log rank test were applied to evaluate PFS rate. Cox proportional risk models were used for univariate and multivariate analyses to identify indicators affecting prognosis. The above statistical analyses were performed using the SPSS 26.0 software, and a two-tailed *P* < 0.05 was considered statistically significant.

## Results

### Baseline characteristics of all patients

**FIGURE 1:**
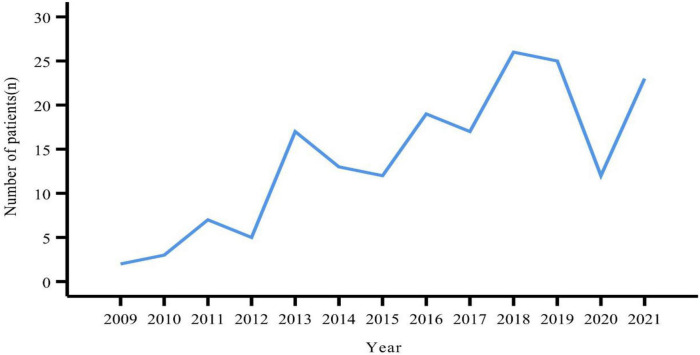
Growing trends in the number of patients with pNENs from 2009 to 2021.

A total of 181 patients were eventually included in this study. The number of patients peaked in 2018 (*n* = 26), followed by 2019 (*n* = 25) (Figure 1). There were slightly more females (*n* = 96, 53.04%) than males (*n* = 85, 46.96%). The age of the patients ranged from 12 to 83 years, with a median age of 51 years. Slightly more patients were from urban than rural areas (53.59% vs. 46.41%). BMI values were obtained for a total of 168 patients, with a median value of 23.60 kg/m^2^. More than half of the patients (*n* = 86, 51.19%) had a BMI within the normal reference range. Smokers and alcohol drinkers accounted for 12.15 and 7.73% of all patients, respectively (Table 1).

**TABLE 1:** Baseline characteristics of 181 patients with pNENs.

Parameters	Total (*n* = 181)
Age (years)	51 (43.5, 58)
**Gender, n (%)**	
Male	85 (46.96%)
Female	96 (53.04%)
**Residence, n (%)**	
Urban areas	97 (53.59%)
Rural areas	84 (46.41%)
BMI (kg/m^2^)	23.6 (21.50, 26.32)
BMI subgroup (n, available)	168
≤18.4	10 (5.95%)
18.5∼23.9	86 (51.19%)
24∼27.9	42 (25.00%)
≥28	30 (17.86%)
**Smoking, n (%)**	
Yes	22 (12.15%)
No	159 (87.85%)
**Drinking, n (%)**	
Yes	14 (7.73%)
No	167 (92.27%)
Size (n, available)	169
>2 cm	78 (46.15%)
≤2 cm	91 (53.85%)
Location (n, available)	172
Head or neck	84 (48.84%)
Body or tail	88 (51.16%)
**Subtype**	
Functional	86 (47.51%)
Non-functional	95 (52.49%)
Grade (n, available)	160
G1	91 (56.88%)
G2 + G3	69 (43.13%)
Stage (n, available)	171
I	63 (36.84%)
II	85 (48.71%)
III	3 (1.75%)
IV	20 (11.70%)
**Progress, n (%)**	
Yes	37 (20.44%)
No	144 (79.56%)

The median tumor size was 2 cm, and 46.15% of the tumors were > 2 cm in size. Non-functional tumors were more frequent than functional tumors (52.49% vs. 47.51%). In addition, highly differentiated G1 tumors were the most frequent, with 91 cases, followed by medium and low differentiated G2 and G3 tumors. According to TNM staging criteria, we counted the tumor stages of 171 patients, among which, 63 (36.84%) were stage I, 85 (48.71%) were stage II, 3 (1.75%) were stage III, and 20 (11.70%) were stage IV (Table 1).

### Preoperative prognostic nutritional index and clinicopathological parameters

**FIGURE 2:**
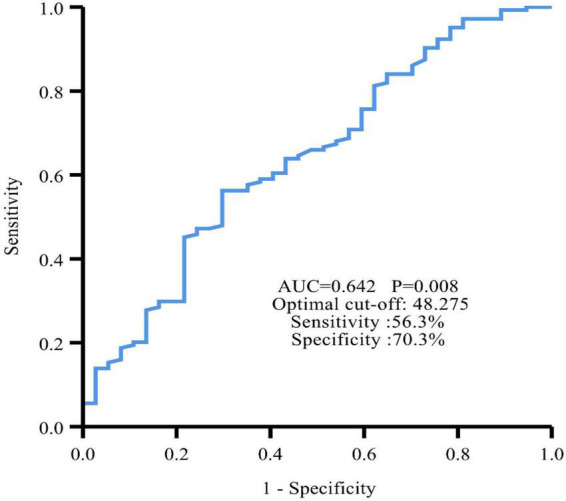
Time-dependent ROC curve revealed that the best cut-off value of PNI to predict PFS was 48.275 with 56.3% sensitivity and 70.3% specificity.

The ROC curve showed an area under the curve (AUC) of 0.642 (95% CI: 0.540∼0.743, *P* = 0.008) (Figure 2). The optimal cut-off value for PNI was 48.275, which was used to divide all patients into a high PNI group (PNI > 48.275) and a low PNI group (PNI ≤ 48.275).

There were slightly more people in the high PNI group than in the low PNI group (*n* = 92 vs. *n* = 89). In both groups, there were more women than men. The gender difference between the two groups was statistically insignificant (*P* = 0.720). Patients in the low PNI group were significantly older than those in the high PNI group (54.64 ± 11.05 years vs. 47.20 ± 14.14 years, *P* < 0.001), and the BMI was similar in both groups (*P* = 0.648). The proportion of smokers (*P* = 0.018) and alcohol drinkers (*P* = 0.022) was significantly higher in the low PNI group compared with that in the high PNI group. Although there were mostly non-functional tumors in the high PNI group (56.52%) and functional tumors in the low PNI group (51.69%), the difference between the two groups was statistically insignificant (*P* = 0.269). The proportion of tumors size > 2 cm was higher in the low PNI group than in the high PNI group (*P* = 0.004). No significant differences were observed between the two groups based on location, grading and staging of tumors (*P* > 0.05).

In terms of routine blood tests and blood biochemical indices, the high PNI group had significantly higher levels of hemoglobin (*P* < 0.001), total and percentage of peripheral blood lymphocytes (*P* < 0.001), and a significantly higher level of serum total protein (*P* < 0.001), serum albumin (*P* < 0.001), triglycerides (*P* = 0.014), and cholesterol (*P* = 0.039) (Table 2).

**TABLE 2:** Associations of PNI with clinicopathologic characteristics of patients with pNENs.

Parameters	High PNI (*n* = 92)	Low PNI (*n* = 89)	*P*-value
Age (years)	47.20 ± 14.14	54.64 ± 11.05	<0.001
Gender, n (%)			0.720
Male	42 (45.65%)	43 (48.31%)	
Female	50 (54.35%)	46 (51.69%)	
Residence, n (%)			0.613
Urban areas	51 (55.43%)	46 (51.69%)	
Rural areas	41 (44.57%)	43 (48.31%)	
BMI (kg/m^2^)	24.07 ± 3.37	23.82 ± 3.93	0.648
BMI subgroup (n, available)	87	81	0.573
≤18.4	5 (5.75%)	5 (6.17%)	
18.5∼23.9	42 (48.28%)	44 (54.32%)	
24∼27.9	25 (28.74%)	17 (20.99%)	
≥28	15 (17.24%)	15 (18.52%)	
Smoking, n (%)			0.018
Yes	6 (6.52%)	16 (17.98%)	
No	86 (93.48%)	73 (82.02%)	
Drinking, n (%)			0.022
Yes	3 (3.26%)	11 (12.36%)	
No	89 (96.74%)	78 (87.64%)	
Size (n, available)	87	82	0.004
>2 cm	40 (45.98%)	38 (46.34%)	
≤2 cm	47 (54.02%)	44 (53.66%)	
Location (n, available)	87	85	0.645
Head or neck	44 (50.57%)	40 (47.06%)	
Body or tail	43 (49.43%)	45 (52.94%)	
Subtype			0.269
Functional	40 (43.48%)	46 (51.69%)	
Non-functional	52 (56.52%)	43 (48.31%)	
Grade (n, available)	83	77	0.482
G1	45 (54.22%)	46 (59.74%)	
G2 + G3	38 (45.78%)	31 (40.26%)	
Stage (n, available)	87	84	0.327
I	34 (39.08%)	29 (34.52%)	
II	44 (50.57%)	41 (48.81%)	
III	1 (1.15%)	2 (2.38%)	
IV	8 (9.20%)	12 (14.29%)	
Progress, n (%)			0.004
Yes	11 (11.96%)	26 (29.21%)	
No	81 (88.04%)	63 (70.79%)	
Hemoglobin (g/L)	133.68 ± 16.21	123.47 ± 16.76	<0.001
Total lymphocyte count (*10^9^/L)	1.97 ± 0.59	1.43 ± 0.52	<0.001
Lymphocytes% (%)	32.77 ± 8.80	26.54 ± 10.45	<0.001
Albumin (g/L)	42.77 ± 2.88	37.10 (35.45, 39.40)	<0.001
Total protein (g/L)	68.59 ± 5.15	62.12 ± 4.54	<0.001
Triglycerides (mmol/L)	1.17 (0.86, 2.10)	1.04 (0.74, 1.36)	0.014
Cholesterol (mmol/L)	4.46 ± 1.03	4.24 (3.57, 4.73)	0.039
LDL-c (mmol/L)	2.56 ± 0.80	2.43 ± 0.66	0.271
HDL-c (mmol/L)	1.2 (0.99, 1.46)	1.1 (0.9, 1.44)	0.118

### Correlations

The preoperative PNI negatively correlated with age (β = −0.226, *P* = 0.004) and cholesterol (β = −0.653, *P* = 0.002) and positively correlated with hemoglobin (β = 0.314, *P* < 0.001), triglycerides (β = 0.351, *P* < 0.001), low-density lipoprotein cholesterol (LDL-c) (β = 0.474, *P* = 0.007), and high-density lipoprotein cholesterol (HDL-c) (β = 0.290, *P* = 0.007) (Table 3).

**TABLE 3:** Multiple linear regression analysis of the correlation between PNI and clinicopathological parameters.

Parameters	β	*P*-value
Age	−0.226	0.004
BMI	0.023	0.766
Hemoglobin	0.314	<0.001
Triglycerides	0.351	<0.001
Cholesterol	−0.653	0.002
LDL-c	0.474	0.007
HDL-c	0.290	0.007

### Preoperative prognostic nutritional index and prognosis

**FIGURE 3:**
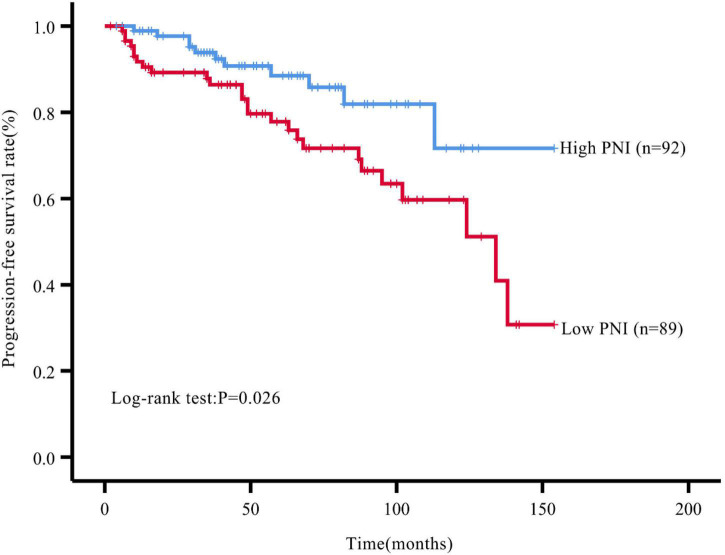
Kaplan–Meier survival curve analysis of PFS rate grouped by PNI levels.

During the follow-up, we found a total of 37 patients (20.44%) with postoperative tumor progression (Table 1). The post-surgery percentage of tumor progression was significantly higher in the low PNI group than in the high PNI group (29.21% vs. 11.96%, *P* = 0.004) (Table 2), and the result of the Kaplan-Meier survival analysis confirmed this result (*P* = 0.026) (Figure 3). The univariate Cox regression analysis showed a significant association between PFS and preoperative PNI, age, tumor functionality, pathological grade, lymph node status, and distant metastasis. Among these, the patients’ post-surgery hazard ratio (HR) for tumor progression in the low PNI group to those in the high PNI group was 2.197 (95% CI: 1.078∼4.477, *P* = 0.030). Next, by incorporating the above statistically different indicators into a multivariate Cox regression analysis, we found that the preoperative PNI (HR: 2.727, 95% CI: 1.174–6.333), tumor functionality (HR: 3.117, 95% CI: 1.0446∼9.305), pathological grade (HR: 5.541, 95% CI: 1.922∼15.974), and lymph node status (HR: 3.959, 95% CI: 1.156∼13.561) were independent predictors of PFS (Table 4). Thus, even after adjusting for confounding factors, the post-surgery risk of tumor progression was significantly higher in the low PNI group compared with that in the high PNI group.

**TABLE 4:** Univariate and multivariate analysis of PFS in patients with pNENs.

Variables	Univariate			Multivariate		
	HR	95%CI	*P*-value	HR	95%CI	*P*-value
PNI (low vs. high)	2.197	1.078∼4.477	0.030	2.727	1.174∼6.333	0.020
Age (>51 vs. ≤ 51)	4.405	1.988∼9.708	0.000			
Subtype (Non-functional vs. functional)	5.717	2.415∼13.536	0.000	3.117	1.044∼9.305	0.042
Grade (G2, G3 vs. G1)	7.970	3.455∼18.381	0.000	5.541	1.922∼15.974	0.002
Lymph node metastasis (yes vs. no)	6.219	2.112∼18.309	0.001	3.959	1.156∼13.561	0.028
Distant metastasis (yes vs. no)	5.000	2.535∼9.860	0.000			

## Discussion

pNENs are the second most common epithelial malignancies of the pancreas (25). Although they are still rare diseases, their incidence is gradually increasing worldwide. Gender differences in the incidence of pNENs are influenced by race, region, and other factors. In the American population, more men than women were reported to have these diseases (3), while the opposite finding was reported in the Italian population (26). The tumors’ high heterogeneity allows for significant differences in individual prognosis, which is also the case for patients with pNENs undergoing surgery. Therefore, it is essential to find appropriate preoperative prognostic predictors.

Due to inappropriate secretion of hormones, patients with pNENs can suffer from malabsorption, abdominal pain, diarrhea, and other gastrointestinal symptoms that lead to a state of malnutrition (27). For tumor patients undergoing surgery, the preoperative nutritional status has almost become an accepted factor affecting the patient’s postoperative prognosis (28). However, in recent years, scholars have mostly focused their prognosis for patients on the impact of tumor cells or the tumor microenvironment, such as CD47 expression and CD163^+^ macrophages (29), cytokeratin-19 (CK-19) (30), and tumor-infiltrating platelets (31). Few studies have assessed whether the preoperative nutritional status has a predictive value in the postoperative progression of patients with pNENs.

PNI was originally proposed by Onodera et al. and used in the prognostic assessment of patients undergoing gastrointestinal surgery (32). In recent years, the PNI has been widely used in clinical practice, with current research focusing on investigating the relationship between the PNI and tumor prognosis. Cadwell et al. found that in elderly cancer patients, PNI was associated with 6-month postoperative mortality (33). In addition, the PNI can be combined with the neutrophil/lymphocyte ratio (NLR), and lactate dehydrogenase (LDH) to synergistically assess PFS and OS in patients with advanced cancer, receiving immunotherapy (34, 35). It has also been reported that the PNI has a significant association with prognosis in patients undergoing surgery and who have digestive system tumors, such as liver and gastric cancer (36, 37). The PNI levels are determined by serum albumin and blood lymphocyte count. Previous studies have shown that serum albumin levels can be used alone to assess the development of several malignancies and to predict the prognosis of patients (38, 39) as it can directly reflect the nutritional status of an organism. Lymphocytes play an important role in tumor immune surveillance and are closely related to the occurrence and development of tumors (40). The total peripheral blood lymphocyte count can reflect the immune function of the body. This retrospective study is the first to use the PNI to reflect the nutritional status of patients with pNENs and fills a gap in the field of research on the predictive value of preoperative PNI for the postoperative progression of pNENs.

In this study, we first determined the optimal cut-off value for the preoperative PNI at 48.275. By analyzing the differences in clinicopathological characteristics between patients in the low PNI and high PNI groups, we found that patients in the low PNI group have a relatively poor baseline condition, which is particularly evident with old age. This result was consistent with most studies (17, 19, 41). Not only were albumin levels and the total peripheral blood lymphocyte count lower in the low PNI group, but also hemoglobin levels, lymphocyte percentage, total protein, triglycerides, and cholesterol, which can also indirectly reflect the nutritional and immune status of the body. BMI is calculated based on weight and height, which has been used as a measure of human nutritional status since the 1990s. Since then, in order to harmonize international terminology, the European Society of Clinical Nutrition and Metabolism (ESPEN) issued a statement in 2015 stating that malnutrition should be diagnosed based on BMI (42). The existing studies found that the relationship between BMI and PNI still seems to be contradictory. For example, the study by Park et al. found a positive correlation between preoperative BMI and PNI (43), while the present study, in agreement with the study by He et al. did not observe a correlation between the two (44), which may be related to the different diseases studied and the differences in the populations included, but the exact reasons for this still need further study with large sample data. In addition, our study also found that patients in the low PNI group had a relatively higher likelihood of tumor progression after surgery compared to the high PNI group. The Kaplan-Meier curve also showed a significantly higher PFS after surgery in the high PNI group compared with that in the low PNI group. The preoperative PNI negatively correlated with age, however, after adjusting for many confounding factors, the preoperative PNI was indeed a significant predictor of postoperative tumor progression. This has been confirmed in other tumors of the pancreas, such as pancreatic ductal adenocarcinoma (41) and pancreatic solid pseudopapillary tumor (45). However, to our knowledge, this is the first time it has been confirmed in pancreatic neuroendocrine tumors.

There is no doubt that our study is a retrospective single-center study, and although it can relatively illustrate our point, it still has limitations. Therefore, our existing findings require further validation by prospective multicenter studies.

## Conclusion

In conclusion, this study shows for the first time that preoperative PNI has a predictive value for postoperative progression in patients with pNENs, and that a low PNI is an independent risk factor of PFS. For patients who undergo surgery, attention should be paid to their nutritional and immune statuses before surgery, and an extra attention should be paid to the close follow-up of patients with a low PNI after surgery.

## Data availability statement

The original contributions presented in this study are included in the article/supplementary material, further inquiries can be directed to the corresponding author/s.

## Ethics statement

The studies involving human participants were reviewed and approved by the Union Hospital, Tongji Medical College, Huazhong University of Science and Technology. Written informed consent from the participants’ legal guardian/next of kin was not required to participate in this study in accordance with the national legislation and the institutional requirements.

## Author contributions

HS conceived the study. MF and LYu contributed to the concept and design. LYa, YC, XC, and QH contributed to the data collect, analysis, and interpretation. MF drafted the manuscript. All authors were involved in the revision of the manuscript and approved the submitted version.
